# Exposure, vulnerability, and resiliency of French Polynesian coral reefs to environmental disturbances

**DOI:** 10.1038/s41598-018-38228-5

**Published:** 2019-01-31

**Authors:** Julie Vercelloni, Mohsen Kayal, Yannick Chancerelle, Serge Planes

**Affiliations:** 1 École pratique des hautes études, PSL Research University, UPVD, CNRS, USR 3278 CRIOBE, BP 1013, 98729 Papetoai, Moorea French Polynesia; 2 Australian Research Council Centre of Excellence for Coral Reef Studies, School of Biological Sciences, The University of Queensland, St Lucia, QLD 4072 Australia; 3 Centre de Formation et de Recherche sur les Environnements Méditerranéens, UPVD, CNRS, UMR 5110, 52 Avenue Paul Alduy, 66860 Perpignan, France; 4 Centre de Recherche sur les Ecosystèmes Marins, Impasse du solarium, 66420 Port-Barcarès, France; 5 Laboratoire d’Excellence “CORAIL”, Université de Perpignan, 52 Avenue Paul Alduy, 66860 Perpignan, France

## Abstract

Preserving coral reef resilience is a major challenge in the Anthropocene, yet recent studies demonstrate failures of reef recovery from disturbance, globally. The wide and vigorous outer-reef system of French Polynesia presents a rare opportunity to assess ecosystem resilience to disturbances at a large-scale equivalent to the size of Europe. In this purpose, we analysed long-term data on coral community dynamics and combine the mixed-effects regression framework with a set of functional response models to evaluate coral recovery trajectories. Analyses of 14 years data across 17 reefs allowed estimating impacts of a cyclone, bleaching event and crown-of-thorns starfish outbreak, which generated divergence and asynchrony in coral community trajectory. We evaluated reef resilience by quantifying levels of exposure, degrees of vulnerability, and descriptors of recovery of coral communities in the face of disturbances. Our results show an outstanding rate of coral recovery, with a systematic return to the pre-disturbance state within only 5 to 10 years. Differences in the impacts of disturbances among reefs and in the levels of vulnerability of coral taxa to these events resulted in diverse recovery patterns. The consistent recovery of coral communities, and convergence toward pre-disturbance community structures, reveals that the processes that regulate ecosystem recovery still prevail in French Polynesia.

## Introduction

Within few decades, the concept of Earth’s ecological vulnerability has arisen and established as an undeniable global issue^1–3^. To understand and offset this trend, increasing efforts have been dedicated to assessing ecosystem health and trajectory by evaluating how exposed, vulnerable, and resilient are natural communities to disturbances, in our changing environment. However, comprehending ecosystem dynamics is not an easy task, given non-linearity in species trajectories, and complex interactions among underlying processes that regulate ecological communities and associated ecosystem functions^4–7^. Such complexities result particularly from the combination of the diversity in species life histories, environmental heterogeneity, and unpredictable impacts of disturbances^8,9^. Consequently, assessing species trajectories is even more challenging in productive and biodiverse ecosystems where species interactions are often strong and complex, and in oscillating environments where disturbances can produce cascading effects and runaway ecosystem collapses^10–12^.

Coral reefs are emblematic examples of biodiverse non-equilibrium ecosystems where community dynamics naturally alternate between demographic expansions in a limited-resource environment, and pulse occurrences of mass-mortality events^10,13–15^.In absence of major disturbance, occupation of habitats and community organization are mostly determined by distribution in primary resources and abiotic forcing, biotic interactions, and availability in refuges from different stresses^5,16^. By periodically decreasing crowdedness, disturbances release ecological niches through availability in space, light, and other limiting vital resources that are redistributed among species via recolonization of habitats, competition, and eventually ecological successions^10,17,18^. Persistence of such non-equilibrium ecosystems relies on the process of recovery, which, if successful, guarantees maintenance in species compositions and the respective ecological functions they fulfil^13,19–21^. However, escalating human-driven alteration of natural habitats and changes in the type and regime of disturbances (notably associated to global climate change) have increasingly weakened species recovery and come to challenge the historical resiliency of ecosystems^1–3,22,23^. In the coastal tropics, this has resulted in declines in the extent of coral reef habitats, and decreases in the ecological quality of many of the remaining reefs which further exacerbates alterations in community organization, loss of ecological functions, and erosion of reef diversity and productivity^24–26^. For example, the Australian’s Great Barrier Reef has been subject to declines in water quality^27^, major outbreaks of the coral predator crown-of-thorns starfish (COTS)^28^ and intense cyclones^29^ as well as unprecedented marine heatwaves^30,31^ whose cumulative effects have come to undermine the historical resiliency of these reefs^23,32–35^. This global demise of Nature is urging for investigations on the directionality and steadiness of community recovery, which can constitute a baseline for future management strategies. This endeavour is particularly fundamental for foundation species such as corals that design ecological habitats, and vulnerable non-equilibrium ecosystems such as tropical coral reefs which are exposed to climate change and disturbances and prone to abrupt ecological shifts^7,11,13,14,20,21,23,36^.

**Figure 1:**
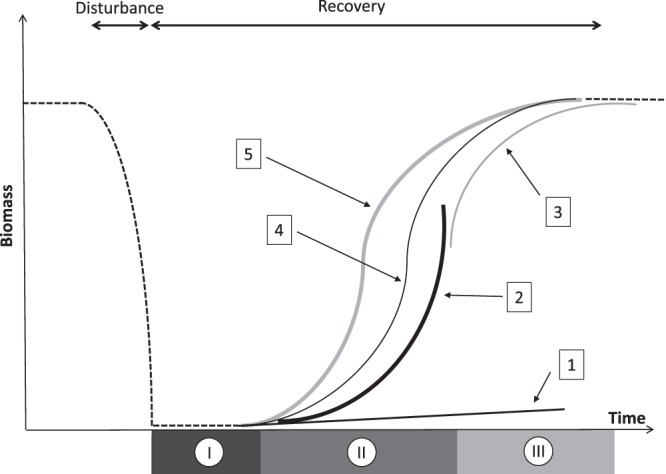
A general theoretical disturbance-recovery model describing the resilience of complex natural ecosystems following disturbances in a limiting environment. The sigmoid recovery response encompasses (I) a linear latency phase of slow recolonization of habitats, (II) an exponential acceleration phase of increasing growth through the utilization of resources in a non-restrictive environment, and (III) a logarithmic deceleration phase of decreasing growth under escalating environmental limitations until reaching an asymptotic saturation at the carrying capacity threshold. This theoretical recovery model can be partially or fully represented mathematically by five functional models: from 1–5 representing, respectively, Linear, Exponential, Logarithmic, Logistic and Gompertz models (see equations in Methods).

Recovery dynamics can be modelled using functional response curves, or functional models^4^, that describe various shapes of population growth^19,37,38^. The parameters estimated from such models constitute descriptors of species demography that express the directionality and kinetics of change during the recovery process, and thus can be used to evaluate resiliency^34,39^. In theory, the recovery process follows a sigmoid trajectory^34^, because populations are expected to show first an accelerating demography after the release of space and limiting resources by disturbances, until their expansion is increasingly slow down by competition when approaching the carrying capacity of the system (Fig. 1). However, empirical observations do not always support this theoretical description, and in many instances, records of species trajectories do not cover the entire process of recovery. This is particularly the case for slow-growing, long-lived organisms that engineer ecological habitats such as reef corals^7,15,38,39^.

Despite their importance for developing conservation strategies, quantifying factors that facilitate coral resilience has revealed being a difficult task, given the high proportion of worldwide reefs that have already undergone consequent changes in their ecological environment^26,28,33,35^. In this context, long-term observations from the disturbance-driven French Polynesian outer-reefs provide a unique opportunity to evaluate coral resilience at large spatial scale^15,40^.

By confronting long-term responses of coral communities to disturbances with functional models describing theory of non-equilibrium ecosystems, we evaluate the level of exposure and the degree of vulnerability of French Polynesian reefs to disturbances, and discuss key ecological processes that contributed to their successful and rapid recovery. Our approach facilitates forecasting forthcoming species dynamics based on the trajectories observed at the earlier stages of recovery, and thus should widely benefit to the preservation of ecosystems despite the current global environmental changes.

## Methods

### Sampling design and spatio-temporal scope

**Figure 2:**
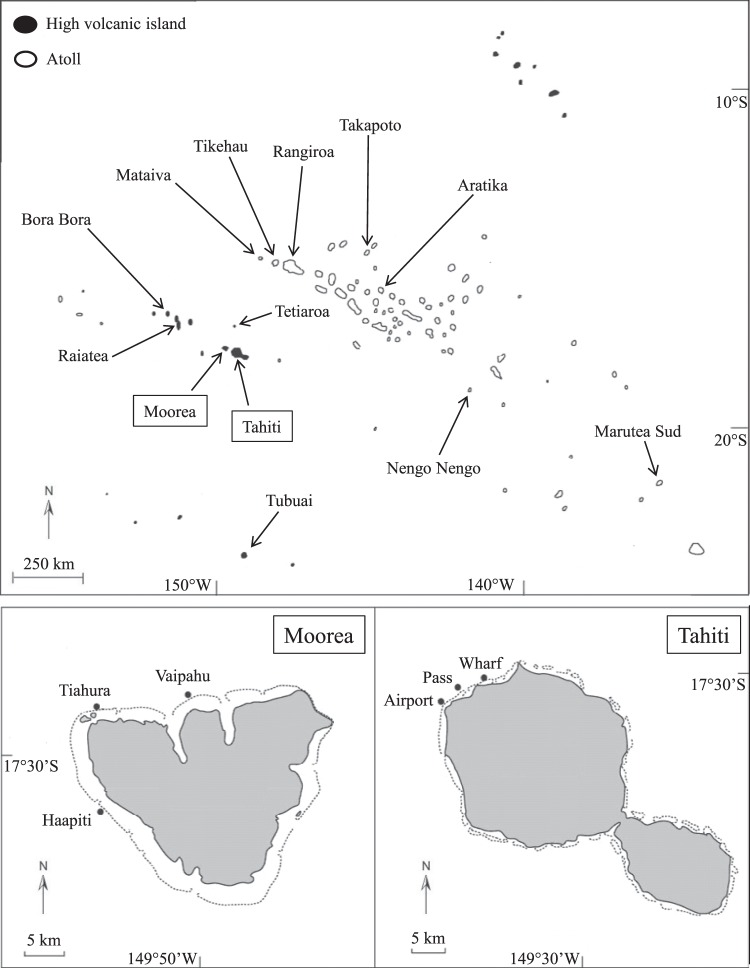
Geographical locations of the monitored reefs. Modified from Adjeroud *et al*. 2005^42^.

From 1994 to 2008, the Polynesia-Mana long-term monitoring program (a local application of the Global Coral Reef Monitoring Network – GCRMN) surveyed benthic communities on 17 reefs located on 13 different islands across French Polynesia, five high-volcanic and eight atolls, encompassing four archipelagos and a wide geographical scale spreading over 15° of longitude and 10° of latitude (Fig. 2). These observations were performed on the outer-reef slopes where coral biomass, diversity, and susceptibility to large-scale disturbances (namely, bleaching events, cyclones, and COTS outbreaks) are maximal^11,40,41^. The sampling design consists in the identification of the sessile organisms lying underneath 1,620 permanent points whose positions are defined by a 10cm-mesh grid within 20 replicate 1 m^2^ photo-quadrats aligned consecutively along the 10 m iso-depth (see^40,42^). Coral taxa are identified at the genus level and coral cover is calculated as the proportion of points occupied by live coral. In the present study, coral cover is calculated by pooling observations over 5 m^2^ of reef area (405 observations performed in five consecutive 1 m^2^ quadrats), providing four replicate measurements per sampling (see Electronic Supplementary Material).

Each reef was surveyed every 2 ± 2 years starting in 1994. As the Polynesia-Mana long-term monitoring program has been modified since 2008, this study is restricted to the first 14 years of coral cover data, therefore excluding the recent occurrence of a new cycle of large-scale disturbances^11^ and subsequent reef recovery^15,21^.

### Disturbance impacts and models of coral recovery

To characterize coral dynamics and compare recovery trajectories, we identified the reefs that had suffered considerable coral mortality and those that had shown substantial recuperation following criteria used during previous investigations. As such, occurrence of a major ecological disturbance was defined as a statistically significant decline in coral cover between consecutive sampling with a loss of at least 33% of the pre-disturbance value^38,39,42^. Similarly, recuperation was defined as a period of significant increase in coral cover following a disturbance, with over the whole period a regain of at least 50% of the cover lost to the disturbance. A recuperation of 100% of the pre-disturbance coral cover was referred to as recovery, the recovery process referring to the entire time period of recuperation from the occurrence of the disturbance until reaching a stable maximum threshold in community size (Fig. 1). Within reef differences in coral cover values among years were evaluated using the nonparametric test of Friedman, and the Wilcoxon test was used *a posteriori*.

Reefs that showed considerable loss in coral cover were used to evaluate impacts of different types of disturbances (namely, bleaching events, cyclones, and COTS outbreaks). For each disturbance, the proportional decline in coral cover expressed in percentage *%loss* was calculated for the entire coral community as well as for each of the three major coral genera, *Acropora*, *Pocillopora*, and *Porites*. Differences in coral *%loss* among different types of disturbances were tested using Kruskal-Wallis. The two-way design Scheirer-Ray-Hare test was used to test for difference in *%loss* among disturbances, coral genera, and their interactions, and the Steel-Dwass test was used *a posteriori* when a significant difference was detected.

Reefs that showed substantial recuperation following disturbances were used to compare the recovery trajectories of corals. Coral recovery trajectories, in terms of total community cover, individual genus cover, and relative contribution of genera within communities, were modelled using functional models describing different shapes of population growth as predicted for the recovery of complex natural ecosystems (Fig. 1). This theoretical recovery model can be partially or fully represented by the five following functional models where the population or community size (*y*) is modelled as a function of time (*t*):

1 $$-\,{\rm{Linear}}\,{\rm{model}}:\,y(t)=b+a\times t$$

2 $$-\,{\rm{Exponential}}\,{\rm{model}}:\,y(t)=b\times {e}^{a\times t}$$

3 $$-\,{\rm{Logarithmic}}\,{\rm{model}}:\,y(t)=\alpha \times (1-{e}^{-a\times t})$$

4 $$-\,{\rm{Logistic}}\,{\rm{symmetric}}\,{\rm{sigmoid}}\,{\rm{model}}:\,y(t)=\frac{\alpha }{1+{e}^{\frac{\beta -t}{b}}}$$

5 $$-\,{\rm{Gompertz}}\,{\rm{asymmetric}}\,{\rm{sigmoid}}\,{\rm{model}}:\,y(t)=\alpha \times {e}^{-\beta \times {b}^{t}}$$

where the slope *a* is the linear, exponential, or logarithmic rate of recovery (respectively equations 1–3); the intercept *b* is the value of *y* at the beginning of recovery (equations 1, 2, 4 and 5); the asymptotic parameter *α* is the threshold value of *y* reached at the end of the recovery process (equations 3–5); and the inflection point *β* is the value of *t* at mid-recovery (equations 4 and 5).

Our modelling approach was designed to account for several sources of variability associated with surveys of species trajectories^6^. Goodness-of-fit diagnostics based on the log-likelihood, analysis of deviance and model residual error distributions were used to select the best model formulations of coral recovery trajectory, at the reef scale. To account for within-reef variability, the selected model was refit separately to data from the four replicate 5 m^2^ areas. A non-overlapping of the 95% confidence intervals of model parameters indicated fine-scale (within-reef) heterogeneity in coral trajectories (see Electronic Supplementary Material). In this case, random effects distinguishing the replicate observations were added to the selected models. Random effects were assumed identically and independently distributed. Temporal autocorrelation of order 1 was also tested, and taken into account in the estimation of model parameters when adequate.

The estimated recovery trajectories were compared among reefs and taxa by comparing model parameters between populations sharing similar shapes in recovery dynamics as described by the functional models (equations 1–5). Overlapping of confidence intervals (95% CIs) of model parameters was used as indicative of non-significant difference. Equations (1–5) were also used to project the post-recovery size and structure of communities beyond the observed data. When individual population trajectories did not show a predictable saturation threshold (i.e. equations 1 and 2), populations sizes were projected in time (*t*) using the respective modelling equations until reaching the time of community cover saturation as calculated for each reef (${t}_{saturation}=2\times {\rm{\beta }}$ in equations 4 and 5, Electronic Supplementary Material). The R package nlme^43^ was used to compute the non-linear mixed-effect models, as well as to perform the goodness-of-fit diagnostics and temporal correlation tests via the corAR1 function.

## Results

### Coral dynamics and the role of large-scale disturbances

**Figure 3:**
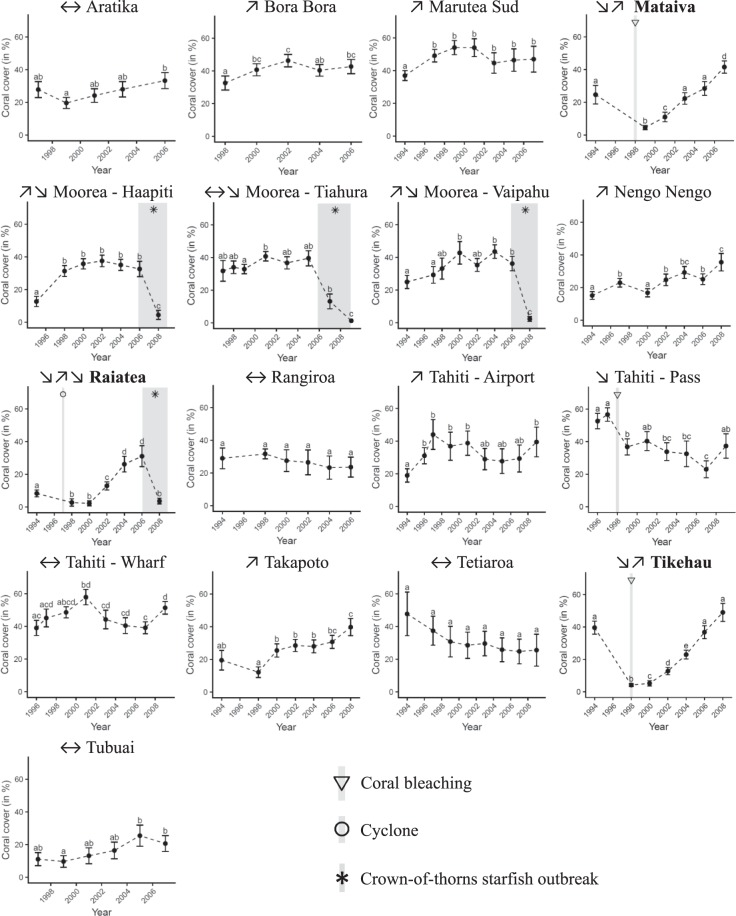
Coral dynamics (mean cover ± 95% confidence intervals) as measured on the 17 reef locations surveyed throughout French Polynesia. Letters on graphs indicate statistically different groups of cover values. Shaded areas on graphs indicate occurrences of major disturbances inducing ≥33% coral mortality: coral bleaching (▽), predator crown-of-thorns starfish outbreak (**✳**), cyclone (○). The general patterns of coral dynamics on each reef is synthesized using arrows: increase (↗), stagnation (↔), decrease (↘). Island names in bold are those where a disturbance-recovery cycle was observed.

French Polynesia coral communities showed diverse trajectories over the period 1994–2008, with reefs exhibiting different directionality in coral cover mostly due to localized impacts of disturbances (Fig. 3). Out of the 17 reef locations from 13 surveyed reefs, five showed relative stability in coral cover, five were recuperating from previous disturbances, and seven underwent at least one of the three major environmental disturbances affecting coral communities in this region. Between March and August 1998, coral mortality associated with mass coral-bleaching events were reported in Mataiva, Tikeau and Tahiti^40,44,45^. These reefs were also impacted by strong waves generated by the cyclone Veli in February 1998 (Mataiva and Tikehau) and cyclones Martin and Osea in November 1997 (Tahiti, *personal communication Y*. *Chancerelle*). The two successive cyclones also induced coral mortality on Raiatea^40^ which later suffered from predatory crown-of-thorns starfish (COTS) outbreak between 2006 and 2010. Similarly, severe coral mortality induced by COTS outbreak was reported in the three surveyed locations on Moorea. There was no consistent trajectory in the directionality of coral dynamics that could be related to the geographical location of reefs or the type of island considered (Figs 2 and 3).

**Figure 4:**
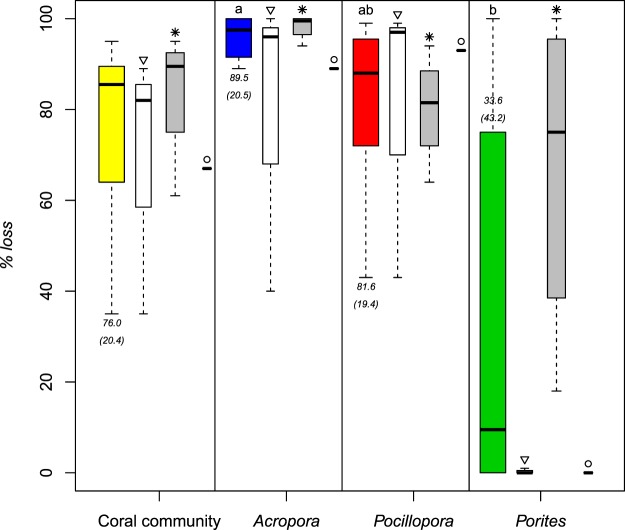
Impacts of the three major environmental disturbances on coral communities and populations of dominant coral genera. For each coral category, box plots from left to right correspond respectively to all disturbances confounded (coloured bars with a different colour code for each group, n_reef_ = 6), coral bleaching (▽, n_reef_ = 3), predator crown-of-thorns starfish outbreak (**✳**, n_reef_ = 2 and 4 reef locations), and cyclone (○, n_reef_ = 1). Boxplots show the distributions of data with medians represented by the tick lines and 95% of the data delimited by the boxes and the minimum and maximum values represented by the thin lines. Letters on graph indicate statistical differences in the susceptibility of coral genera to disturbances. The average percentage of coral loss values (±SE) are also displayed as text.

**Figure 5:**
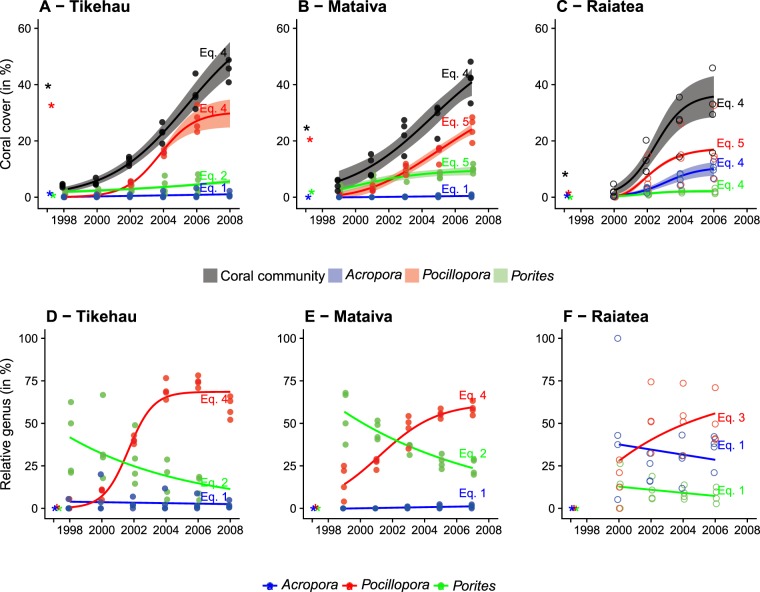
Recovery trajectory of coral communities and populations of dominant coral genera on the three islands where a disturbance-recovery cycle was observed. Panels (**A**–**C**) illustrate recovery in population and community size as expressed by absolute cover. Panels (**D**–**F**) illustrate recovery in community structures as expressed by relative-contribution of populations to communities. Dots indicate observations (filled dots for post-bleaching recovery and hollow dots for cyclone), lines are estimated functional responses and shaded areas show the 95% confidence intervals of the regressions. Note that the panels (**D**–**F**) do not show regression intervals because they were sometimes too wide to be displayed. Asterisks indicate the pre-disturbance sizes (**A**–**C**) and relative-contributions (**D**–**F**) of the different populations and communities. The equation describing each regression is indicated on the graphs (equations 1–5, refer to the core of the manuscript for the mathematical formulae). See Supplementary Materials 2 for the estimated parameters of each equation and the associated *p*-values.

The major disturbances induced severe mortality of corals with in average *%loss* = 76% ± 20.4 standard error SE (Fig. 4). The different types of perturbations resulted in equivalent decline in coral community cover (Kruskal-Wallis, *df* = 2, *p-value* = 0.26). Corals showed taxonomic variability in their susceptibility (Kruskal-Wallis, *df* = 2, *p-value* = 0.02), *Acropora* and *Pocillopora* being most sensitive and consistently undergoing mass mortality facing each disturbance (*%loss*_*Acr*_ = 89.5 ± 20.5 SE and *%loss*_*Poc*_ = 81.6 ± 19.4 SE), and *Porites* being more resistant (*%loss*_*Por*_ = 33.6 ± 43.2 SE). No significant differences were detected in the susceptibility of the three dominant coral genera to the different types of disturbances (Scheirer-Ray-Hare, interaction *Disturbance* × *Genus*, *df* = 4, *p-value* = 0.473), although the taxonomic dominance of communities was temporarily altered by bleaching events on the atolls Tikehau and Mataiva, but not by the cyclones that impacted the high volcanic island of Raiatea (Fig. 5).

### Population and community recovery

Out of the seven reef locations affected by major disturbances, three showed full recovery in terms of coral community cover within the process of this study, while the four remaining locations were impacted by the 2006 disturbance too late within the scope of this study (expanding 1994–2008) to capture recovery (Fig. 3). A return to the pre-disturbance coral cover was estimated within 5 years in Raiatea following a relative decline of *%loss* = 67% (from 8.3% to 2.15%), 7 years in Mataiva following *%loss* = 82% (from 24.6% to 4.6%), and 10 years in Tikehau after *%loss* = 89% (from 39.6% to 4.1%, Electronic Supplementary Material). The five functional response models (Fig. 1) were used to estimate the recovery dynamics of coral populations and communities. Coral communities on the three recovering reefs followed a consistent symmetrical sigmoid trajectory (Fig. 5). Differences in model coefficients however indicated contrasting recovery kinetics among the three islands (Electronic Supplementary Material). Across reefs, the sizes of the coral communities at the end of the recovery process were proportional to their pre-disturbance sizes. An even higher coral cover was estimated at Tikehau with a projected, post-recovery saturation threshold predicted at a cover of 65.4% (asymptotic parameter *α*, ± 7.1% SE) after 17 years (mid-saturation period *β* = 8.4 years ± 0.5 SE, Electronic Supplementary Material). The magnitude of recovery was lowest in Raiatea where the estimated 36.7% saturation threshold was almost reached within 10 years. An intermediate pattern was observed in Mataiva with a saturation threshold in coral cover estimated at 56.9% after 15 years (Fig. 5, Electronic Supplementary Material).

**Figure 6:**
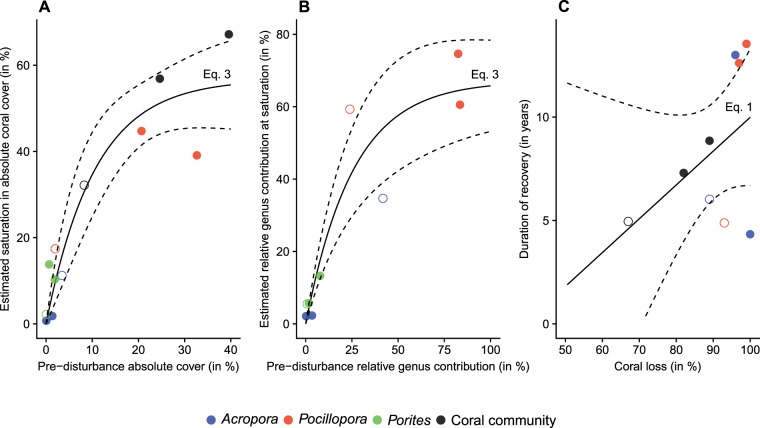
Relationships between the pre-disturbance sizes (**A**) and compositions (**B**) of communities and the estimated values of these variables when reaching saturation in coral community at the end of the recovery process. The panel **C** illustrates the relationship between disturbance intensity (*%loss*) and duration of recovery (time required to reach the pre-disturbance cover value, see Electronic Supplementary Material). Dots indicate observations (filled dots for post-bleaching recovery and hollow dots for cyclone) and lines are functional responses. The equation describing each regression is indicated on the graphs (equations 1–5, refer to the core of the manuscript for the mathematical formulae). See Electronic Supplementary Material for the estimated parameters of each equation and the associated *p*-values.

Coral populations showed contrasting trajectories during the recovery process as described by different functional models and differing model coefficients suggesting different rates of recovery (Fig. 5). *Pocillopora* was most vigorous in recovery and rapidly dominated recuperating communities by following a sigmoid increase (equations 4 and 5). Its populations however showed different recovery rates among reefs, which resulted in contrasting post-recovery saturation thresholds (see estimated asymptotic parameter *α* in Electronic Supplementary Material). *Acropora* and *Porites* populations showed variable dynamics on recovering reefs, with a faster growth of *Porites* on the atolls Tikehau and Mataiva that were impacted by bleaching, and a higher increase of *Acropora* on reefs surrounding the high-volcanic island Raiatea that was impacted by a cyclone. Overall, coral population sizes and community structures consistently converged toward a state that was a function of their pre-disturbance values via a logarithmic relationship (Fig. 6). No significant relationship was found between the severity of disturbances as expressed by coral *%loss*, and the duration of recovery (*p-value* > 0.05; Electronic Supplementary Material).

## Discussion

### Exposure to disturbances

Coral communities in French Polynesia undergo particularly high frequencies of intense disturbances that decimate populations, yet recover within a decade even from major mass mortality events, constituting a singular vibrant coral system (^15^; this study). The reef trajectories illustrate well the theoretical disturbance-recovery pattern characterizing non-equilibrium ecosystems (Fig. 1). Within the temporal scope of this study (1994–2008) conducted at a regional scale, a least one major episode of each of the three most impactful disturbances to coral reefs (bleaching, COTS, and cyclone) was recorded. These disturbances affected 42% of surveyed reefs and differed in term of spatial impacts. As a result, coral dynamics were regionally asynchronous, and many reefs were located at differing positions along the theoretical disturbance-recovery pattern. The COTS outbreak was most widespread and affected 24% of reefs, against 18% affected by the 1998 coral-bleaching event, and 6% by the consecutive cyclones in 1997. These statistics have further increased over recent years, with ongoing propagation of COTS throughout the Society and Australes archipelagos until 2010 and the additional passage of a cyclone near Society Islands in 2010, which resulted in major upheaval of reef communities^11^. Some recent bleaching events were also reported in 2016 and 2017, mainly located in the Tuamotu and Austral archipelagos.

Long-term observations of reef communities around the island of Moorea indicate both COTS outbreaks and cyclones impact reefs in this region with a periodicity of ~20 years, whereas coral-bleaching events occur every ~4 years^11,40,41,46^. However, in contrast with COTS outbreaks and cyclones which consistently result in abrupt coral decline, most bleaching events do not constitute major disturbances to coral communities in French Polynesia, given the selective decimation of few susceptible taxa^46,47^. Similar observations are reported from the Great Barrier Reef, another vast coral system of the South Pacific, where most coral decline has historically been also associated with COTS outbreaks and storms while bleaching events were often less impactful over broad spatial scales^28,35,39^ although this pattern is increasingly challenged after the back-to-back extreme marine heatwaves in 2016 and 2017^30^. However, in contrast with the dynamics observed in French Polynesia, abrupt major disturbance events inducing as much as 33% decline in coral cover have seldom been observed on the Great Barrier Reef where a longer history of observation may be required to capture multiple occurrences of such intense events^14,23,34^. At a broader scale, the 1998 and 2016 coral-bleaching events and COTS outbreaks observed in 2006–2010 in French Polynesia were part of global phenomena^26,48^. However, a cloudy weather above the Society archipelago presumably mitigated the effects of the coral bleaching event in 1998^44^ and possibly 2016. Overall, the French Polynesian reef system appears as a dynamic mosaic of coral communities that follow different trajectories in response to more or less localized environmental disturbances.

### Vulnerability to disturbances

Despite fundamental differences in their nature and pace of action^11,17,40^, the episodes of coral bleaching, COTS outbreak, and cyclone intercepted in our dataset induced equivalent declines of coral communities. The average 76% decline in coral cover attributable to these events was however unequally shared among the dominant coral taxa. With respectively 90% and 82% of decline on average, *Acropora* and *Pocillipora* populations were most sensitive to disturbances and underwent quasi-extirpation at each event. In contrast, *Porites* was more resistant with a decline of 34%. These differences in coral vulnerability to disturbances concord with the contrasting life history characteristics distinguishing the three taxa in terms of colony morphology and porosity of the skeleton, thickness in tissue layer, and palatability for predators, which are major determinants of coral susceptibility to cyclone, bleaching, and COTS^11,49,50^.

### Recovery from disturbances

Despite undergoing a sustained regime of intense disturbances of multiple types, the French Polynesian outer-reef system shows a particularly high resilience capacity, with full recovery in coral cover repeatedly observed within 5–10 years following mass mortality events (^15^, this study). This fast replenishment of communities seems to be at the highest level of recovery achievable for slow growing, habitat-forming organisms such as reef-building corals. A similar pattern is reported on tropical forests where systems that have evolved in more frequently disturbed natural environments also show the highest aptitude for recovery^22^. Comparably, on Australia’s Great Barrier Reef where many coral communities are exposed to different types of disturbances, the shortest coral community recovery periods observed extend 7–10 years, and are reported along with examples of longer duration and failure in recovery^23,28,34^. Reports of full coral recovery from mass mortality events are even scarcer in other regions, and extend beyond the decadal timescale^51^. Importantly, in our broad-scale survey, no major decline without subsequent recovery was observed on any reef, confirming that the coral communities systematically bounce back following mortality events and thus the reefs possess a high resiliency. This high recovery capacity of reefs is shown to be driven by the elevated ability of coral larvae to repopulate reefs shortly after the impacts of disturbances^21,52,53^, which implies a sustained connectivity among populations on spatial scales that extend beyond the range of disturbances. As such, the fragmented insular reef system in French Polynesia can be seen as a network of inter-connected coral communities with asynchronous dynamics, which guarantees an ever preserved stock of adult populations whose reproductive output can provide larvae for replenishment of disturbed reefs^40^ and maintain the extrinsic resistance of these communities to major ecological shifts^36^. Nevertheless, coral decline is of increasing concern on some localized lagoonal reef habitats of French Polynesia that are exposed to higher influence from human populations and follow different dynamics from that of outer-reef coral communities^54^.

Coral communities on the three recovering reefs showed a comparable symmetrical-sigmoid trajectory shape, suggesting that coral replenishment was governed by similar regulatory processes determining habitat colonization and saturation at a large spatial scale. The three reefs, however, recovered at differing rates, each tending toward a distinct saturation threshold that probably varies across reefs^21,55^. Taxonomic differences in recovery trajectories of coral populations concord with the contrasting life history characteristics of these species in French Polynesia. Indeed, coral community recovery was consistently dominated by *Pocillopora* which has the highest reproduction rate and a life strategy promoting a fast recolonization of habitats at a large spatial scale^56^. The sigmoid recovery trajectory, depicting an accelerating expansion of its populations after a relatively short period of latency and a slowing rate of expansion as approaching a saturation threshold concords with findings that suggest a density dependent regulation of recruitment in this taxon^52,57^. *Acropora* and *Porites* showed slower demography with recovery trajectories often restricted to partial representations of the theoretical sigmoid recovery pattern as illustrated in Fig. 2. Interestingly, coral recovery dynamics led to the rise of *Porites* on reefs that were impacted by bleaching, and of *Acropora* following cyclone. Hence, among the dominant coral taxa in French Polynesia, *Porites* is the most resistant to bleaching^40,46,50^, and the high capacity of *Acropora* to propagate through fragmentation confers a strong potential for positive responses to cyclonic events^52^.

Despite differing levels of vulnerability of taxa to disturbances and different history of disturbance on reefs, the recovering coral populations and communities converged toward their pre-disturbed states thus preserving their community abundances and structures. These findings attest of the resiliency of the French Polynesian coral system and contrast with the globally increasing examples of altering coral communities and ecological shifts to alternative stable reef states^26^. The estimated rates of coral recovery on the relatively unaltered French Polynesian outer-reefs can thus constitute a valuable baseline for evaluating reef resilience in other regions and in the future. Our results particularly show that not only French Polynesian outer-reefs still possess strong ecological attractors that keep these ecosystems in a conventional state of coral dominance across disturbance-recovery cycles, but also that coral trajectories on recovering reefs converge to a predictable and preserved community structure regardless of disturbance history and species life history.

### Systematic convergence toward pre-disturbance community structures

Dramatic decline in the quantity and quality of natural ecosystems has drawn much research and conservation efforts toward assessment of ecosystem trajectory and resiliency^1,2,23,24,58^. In this endeavour, both theoretical and empirical approaches have been developed with differing degrees of complexity and over different scales of biological organization^6,15,19,20,22,37^. Yet, a standardized framework that bridges between these different approaches, and provides a common ground that facilitates quantitative understanding of community dynamics and inter-system comparisons, has been lacking. By confronting observed species dynamics with ecological theory of resilient systems, our approach allows estimating persistency in community sizes and composition beyond system fluctuations. In particular, the functional models we provide constitute a flexible and accurate set of tools for modelling specific portions of species trajectories that are of ecological interest, as illustrated here for recovery processes, and should benefit understanding and predicting future community dynamics. Applied to long-term data on reef-building coral dynamics from the frequently disturbed yet resilient French Polynesia reef system, this approach revealed systematic convergence in community recovery trajectories over a broad spatial scale which, we argue, can be used as a measure of ecosystem resistance to ecological shifts. As the frequency and intensity of disturbances are expected to keep increasing with ongoing human pressures and climate change, our approach opens new paths to detecting early signs of failure in community recovery and predicting forthcoming trajectories in coral reefs and other ecosystems.

## Supplementary information

Electronic Supplementary Material

## Data Availability

The authors confirm that all data underlying the findings are fully and freely available under creative commons licence from the Institut national des Sciences de l′Univers. Data are from the Polynesia Mana surveys whose authors may be contacted at joachim.claudet@gmail.com.
