# Supplementary material for: Exposure, vulnerability, and resiliency of French Polynesian coral reefs to environmental disturbances
Source: Sci Rep. 2019 Jan 31;9:1027. doi: 10.1038/s41598-018-38228-5 (PMC6355954; doi:10.1038/s41598-018-38228-5)
Supplement: Supplementary file 1 — Electronic Supplementary Material [file 41598_2018_38228_MOESM1_ESM.pdf]

ELECTRONIC SUPPLEMENTARY MATERIAL

Exposure, vulnerability, and resiliency of French Polynesian coral reefs to environmental disturbances. Vercelloni J, Kayal M, Chancerelle Y and Planes S.

1. Fine-scale coral dynamics

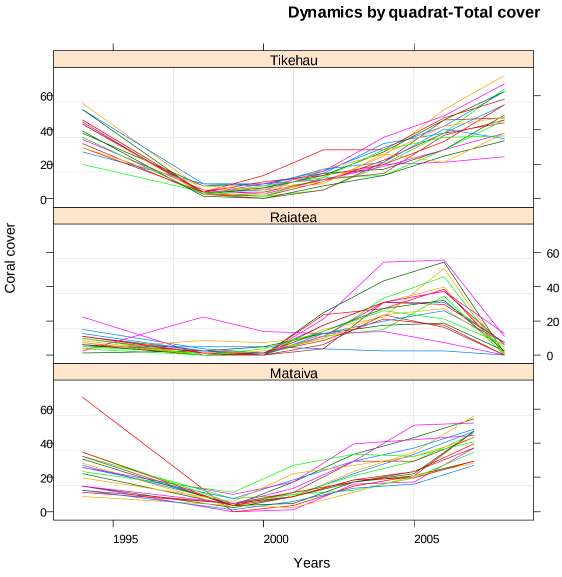

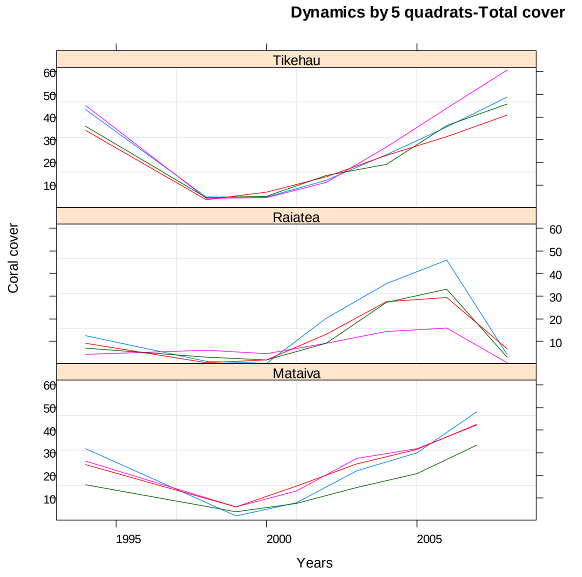

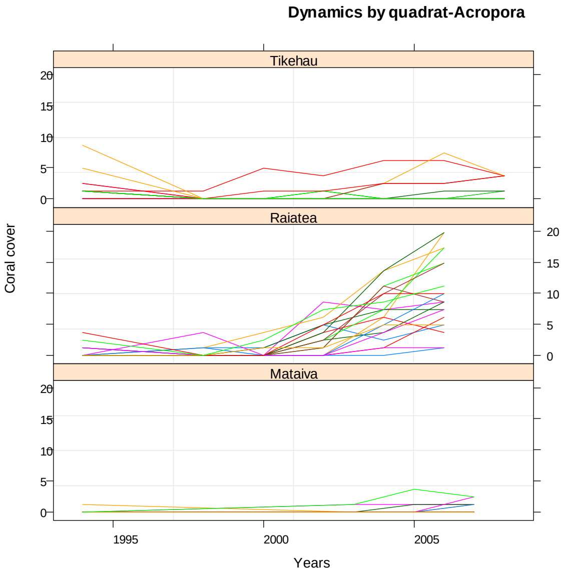

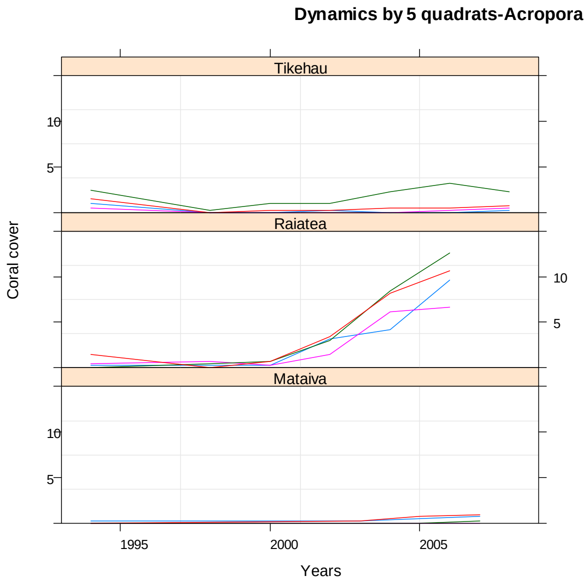

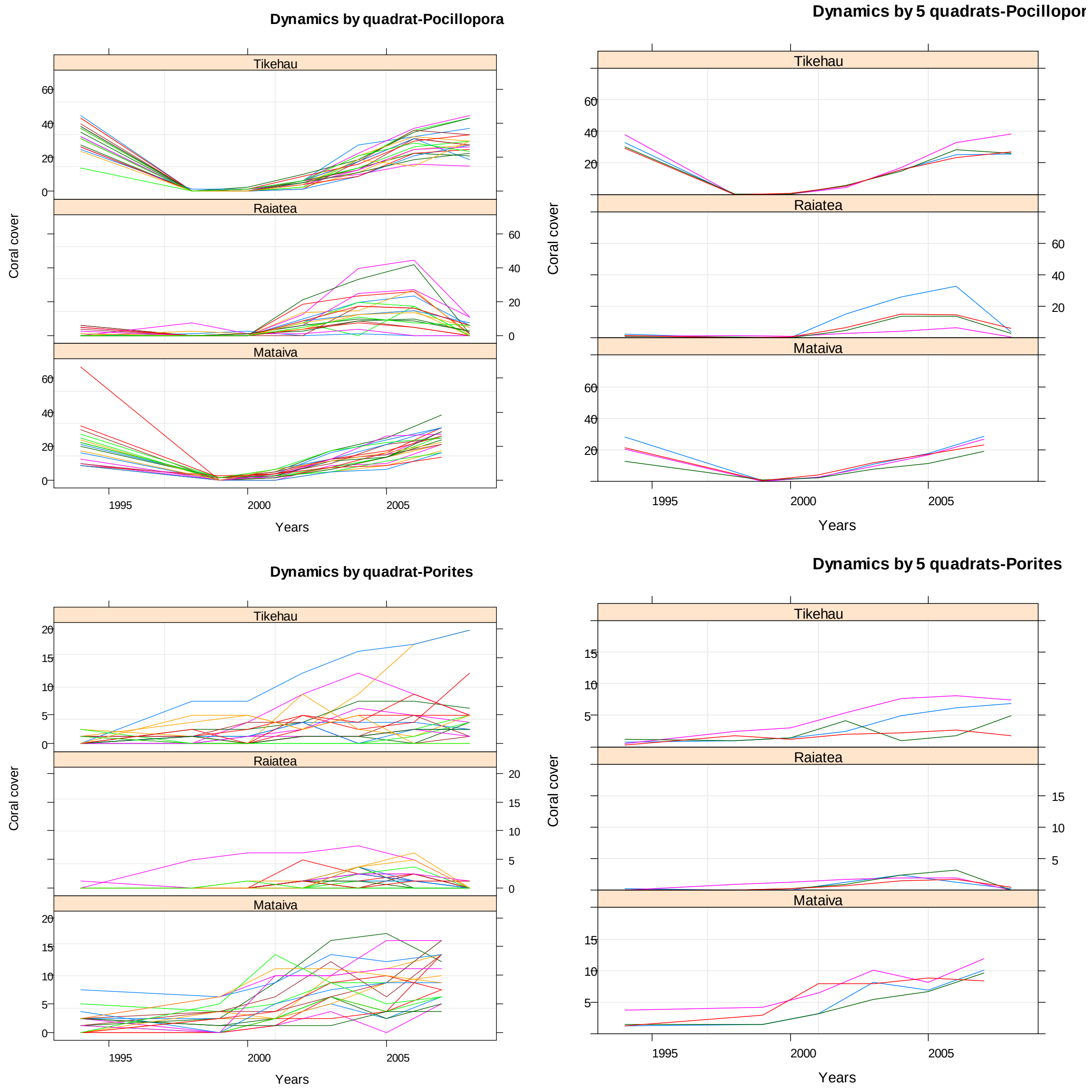

Figure A1 Coral cover dynamics as illustrated per individual 1m^2 quadrat (left) and per combined 5×1m^2 transect (right) at each of the three reefs where a full disturbance-recovery cycle was observed. Trajectories are drawn on separate plots for the entire coral community (total cover) and for each of the three dominant genera (*Acropora*, *Pocillopora*, *Porites*).

2. Calculation of recovery duration

- Linear model: $y(t) = b + a \times t$ (*Eq.1*)

$$t_{recovery} = \frac{y_{pre\_dist} - b}{a}$$

- Exponential model: $y(t) = b \times exp^{a \times t}$ (*Eq.2*)

$$t_{recovery} = \frac{\log(y_{pre\_dist} - b)}{a}$$

- Logarithmic model: $y(t) = \alpha \times (1 - exp^{-a \times t})$ (*Eq.3*)

$$t_{recovery} = -\frac{\log(\frac{y_{pre\_dist}}{\alpha} + 1)}{a}$$

- Logistic symmetric sigmoid model: $y(t) = \frac{\alpha}{1 + exp^{\frac{\beta - t}{b}}}$ (*Eq.4*)

$$t_{recovery} = \beta - b \times \log(\frac{\alpha}{y_{pre\_dist}} - 1)$$

- Gompertz asymmetric sigmoid model: $y(t) = \alpha \times exp^{-\beta \times b^{t}}$ (*Eq.5*)

$$t_{recovery} = \log\frac{-(\log(y_{pre\_dist}) - \log(\alpha))}{b}$$

3. Impacts of disturbance and estimated recovery duration

Table ESM3. Major variations as observed in the coverage (mean % ±Standard Error) of coral communities and dominant populations on the seven reefs affected by disturbances. For those reefs that recovered within the process of this study, the estimated recovery durations are also displayed. The duration of recovery was based on formulas show above.

**Island**		**Pre-disturbance cover**	**Post-disturbance cover**	***Loss* (%)**	**Disturbance**	**Duration of recovery (years)**
**Mataiva**	Coral community	24.63 (2.90)	4.57 (0.64)	82	Bleaching (1998)	6.91
	Acropora	0.06 (0.06)	0	100		4.33
	Pocillopora	20.56 (2.95)	0.62 (0.17)	97		7.47
	Porites	1.91 (0.42)	2.53 (0.49)	-		
Tahiti (Pass)	Coral community	56.67 (2.13)	36.79 (2.52)	35	Bleaching (1998)	
	Acropora	5.93 (2.18)	3.58 (1.28)	40		

	Pocillopora	23.83 (2.50)	13.58 (1.08)	43		
	Porites	7.47 (1.73)	7.28 (1.98)	1		
**Tikehau**	Coral community	39.57 (2.06)	4.14 (0.49)	89	Bleaching (1998)	9.43
	Acropora	1.36 (0.47)	0.06 (0.06)	96		13
	Pocillopora	32.65 (1.75)	0.06 (0.06)	99		9.96
	Porites	0.68 (0.19)	1.54 (0.43)	-		
Moorea (Vaipahu)	Coral community	36.17 (2.25)	1.84 (0.64)	95	COTS (2006-2009)	
	Acropora	15.80 (1.46)	0.06 (0.06)	99		
	Pocillopora	13.52 (1.94)	0.85 (0.58)	94		
	Porites	0.93 (0.62)	0.38 (0.20)	59		
Moorea (Tiahura)	Coral community	13.15 (2.33)	1.29 (0.56)	90	COTS (2006-2009)	
	Acropora	2.1 (0.93)	0	100		
	Pocillopora	5.93 (1.54)	0.99 (0.57)	83		
	Porites	3.02 (0.83)	0	100		
Moorea (Haapiti)	Coral community	32.59 (2.41)	12.82 (2.79)	61	COTS (2006-2009)	
	Acropora	7.41 (1.23)	0.46 (0.31)	94		
	Pocillopora	15.37 (1.71)	5.87 (1.52)	64		
	Porites	3.21 (0.80)	2.65 (0.77)	18		
Raiatea	Coral community	31.05 (3.26)	3.58 (0.89)	89	COTS (2006-2009)	
	Acropora	9.88 (1.35)	0	100		
	Pocillopora	16.79 (2.80)	3.33 (0.84)	80		
	Porites	2.04 (0.40)	0.19 (0.14)	91		
**Raiatea**	Coral community	8.27 (1.08)	2.72 (1.13)	67	Cyclone (1997)	4.32
	Acropora	3.46 (0.23)	0.37 (0.20)	89		5.44
	Pocillopora	1.98 (0.51)	0.56 (0.38)	93		4.76
	Porites	0.06 (0.06)	0.25 (0.25)	-		

4. Details of model estimates

The five functional models that were used in the study:

(1) Linear model: $y(t) = b + a \times t$;

(2) Exponential model: $y(t) = b \times e^{a \times t}$

(3) Logarithmic model: $y(t) = \alpha \times (1 - e^{-a \times t})$

(4) Logistic symmetric sigmoid model: $y(t) = \frac{\alpha}{1+e^{\frac{\beta-t}{b}}}$

(5) Gompertz asymmetric sigmoid model: $y(t) = \alpha \times e^{-\beta \times b^{t}}$

Table ESM4.1 Estimated regression parameters as calculated in modelling the recovery dynamics of corals in Figure 5 and 6. Asterisks indicate significant difference of parameter estimates from zero (* for $p < 0.05$, ** for $p < 0.01$, *** for $p < 0.001$), and grouping symbols (#, $, §, µ) are used to discriminate significantly different coral categories within similar trajectories (*i.e.* similar models with comparable parameters). Coloured cells show parameters estimates that required random effects in the model. Exp. and Log. stand for Exponential and Logarithmic, respectively. Refer to the Methods section in the core of the manuscript for further details on the equations.

Coral cover (figure. 5 graphs a-c)

Reef	Populations	Selected Model	Model parameters and CI							
			A	**CI**	***b***	**CI**	***α***	**CI**	***β***	**CI**
**Tikehau**	Coral com. #	*Logistic*			2.34***	1.93-2.76	65.38 ***	51.51-79.25	8.42***	7.44-9.39
	Acropora	*Linear*	0.09 *0.11*	-0.02- 0.21	-0.01 *0.89*	-0.26 – 0.23				
	Pocillopora	*Logistic*			1.04***	0.99-1.10	30.26 ***	24.72-35.79	6.77 ***	6.46-7.07
	Porites	*Exp.*	0.13**	0.09-0.18	1.49**	0.97-2.12				
**Mataiva**	Coral com. #$	*Logistic*			2.57 **	1.03-4.11	56.89 *0.09*	21.49-92.3	7.60 *0.06*	3.88-11.32
	Acropora	*Linear*	0.06 *	0.02-0.10	-0.20 *0.07*	-0.43- 0.03				
	Pocillopora	*Gompertz*			0.79**	0.67-0.91	44.74 *0.11*	12.91-76.57	6.6 *0.06*	3.29-9.91
	Porites	*Gompertz*			0.67***	0.46-0.88	9.82***	7.15-12.48	2.92*	0.57-5.27
**Raiatea**	Coral com. $	*Logistic*			0.95***	0.65-1.24	36.69***	27.95-45.43	5.48***	5.12-5.84
	Acropora	*Logistic*			1.06**	0.79-1.32	10.69***	7.67-13.71	6.13***	5.75-6.50
	Pocillopora	*Gompertz*			0.44*	0.36-0.51	17.41*	16.59-18.23	51.92 *0.25*	9.53-94.30
	Porites	*Logistic*			0.73 *0.06*	0.04-1.43	2.11***	1.49-2.73	4.47 ***	3.21-5.74

Genera relative contribution (figure 5, graphs d-f)

Reef	Populations	Selected Model	Model parameters and CI							
			A	**CI**	***b***	**CI**	***α***	**CI**	***β***	**CI**
**Tikehau**	*Acropora* §	*Linear*	-0.14 *0.22*	-0.37-0.08	4.06 *0.08*	1.57-5.84				
	Pocillopora	*Logistic*			0.92***	0.78-1.06	74.62***	72.47-76.86	4.83***	4.68-4.99
	Porites µ	*Exp.*	-0.13***	-0.17- -0.08	47.63***	26.41-68.85				

**Mataiva**	*Acropora* §	*Linear*	0.16 **	0.08-0.24	-0.45*0.08*	-0.99- 0.10				
	Pocillopora	*Logistic*			1.69**	1.15-2.62	60.55***	54.21-71.87	4.28 **	3.61-5.29
	Porites µ	*Exp.*	-0.10 ***	-0.15- -0.06	70.73***	51.91-89.55				
**Raiatea**	*Acropora*	*Linear*	0.51*0.51*	-1.71-2.74	22.32*	9.50-35.15				
	Pocillopora	*Log.*	0.91*0.46*	-1.24- 3.08			46.39*	24.60- 68.19		
	Porites	*Linear*	-0.12 *0.92*	-3.97- 3.72	13.32 *0.15*	-8.81-35.43				

**Recovery trajectory (figure 6, graphs a-c)**									
	**Selected Model**	**Model parameters and CI**							
		A	**CI**	***b***	**CI**	***α***	**CI**	***β***	**CI**
**Estimated coral cover**	*Log.*	0.09*	0.03-0.16			56.77***	43.62-69.91		
**Estimated genera contribution**	*Log.*	0.04 0.08	0.001-0.076			67.13***	45.25-89.01		
**Observed duration of recovery**	*Linear*	0.16 0.22	-0.12-0.45	-6.36 0.58	-32.45-19.72				
